# Phylogeny and taxonomy of *Willeya* (*Verrucariales*, *Verrucariaceae*), including two new species and two newly recorded species from China

**DOI:** 10.3897/mycokeys.137.197566

**Published:** 2026-07-20

**Authors:** Xinyi Wang, Yuan Wang, Steven D. Leavitt, Wenzhen Zhu, Xin Zhao

**Affiliations:** 1 College of Agriculture and Biology, Liaocheng University, Liaocheng 252059, China College of Agriculture and Biology, Liaocheng University Liaocheng China; 2 Department of Biology & M.L. Bean Life Science Museum, Brigham Young University, Provo, UT 84602, USA Department of Biology & M.L. Bean Life Science Museum, Brigham Young University Provo United States of America

**Keywords:** Biodiversity, crustose lichens, molecular systematics, morphology

## Abstract

*Willeya* Müll. Arg. (*Verrucariaceae*, *Eurotiomycetes*, *Ascomycota*) is a genus of crustose lichen-forming fungi. To date, no comprehensive taxonomic revision of *Willeya* in China has been conducted. In this study, we performed morphological examinations, multi-gene phylogenetic analyses based on nrITS, mtSSU, and nuLSU sequences and thin-layer chromatography on specimens collected from four Chinese provinces. As a result, two new species, *Willeya bispora* Xin Yi Wang & X. Zhao, *W. guizhouensis* Xin Yi Wang & X. Zhao and two new records, *W. japonica* (B. de Lesd) Gueidan and *W. protrudens* Gueidan are reported from China. Detailed descriptions, illustrations, and comparisons with closely related taxa are provided for all four species. In addition, we discuss all species of *Willeya* previously reported from China. A dichotomous key for the identification of all known *Willeya* species worldwide is also presented.

## Introduction

The genus *Willeya* Müll. Arg. (*Verrucariaceae*, *Eurotiomycetes*, *Ascomycota*) has long been a focal subject of taxonomic debate. Müller (1883) originally established the genus to accommodate *Staurothele diffractella* (Nyl.) Tuck., primarily on the basis that its ascospores remain pale at maturity—a characteristic distinguishing it from *Staurothele* Norman, in which ascospores become dark brown. Later, a second species, *W. rimosa* Müll. Arg., was described from Vietnam (Müller 1889). Clements (1909) subsequently proposed the genus *Phalostauris* Clem. for *Staurothele* with pale ascospores. Despite these early taxonomic treatments, neither *Willeya* nor *Phalostauris* was widely accepted by subsequent lichenologists and both were largely treated as synonyms of *Staurothele* (Thomson 1991; Brodo et al. 2001).

The advent of molecular phylogenetics has greatly refined our understanding of generic delimitations within the *Verrucariaceae* (Gueidan et al. 2007, 2009; Savić and Tibell 2008; Savić et al. 2008; Muggia et al. 2010; Prieto et al. 2010, 2012). Early molecular studies revealed that *Staurothele diffractella* was not closely related to *Staurothele* s.str., but instead formed a sister lineage to *Endocarpon* Hedw., a genus characterized by squamulose to subfruticose thalli (Gueidan et al. 2007, 2009). As a result, *S. diffractella* was initially transferred to *Endocarpon* as *E. diffractellum* (Nyl.) Gueidan and Cl. Roux (Gueidan et al. 2007). However, subsequent molecular analyses demonstrated that this lineage was distinct from *Endocarpon* and comprised a discrete clade of crustose, epilithic species with pale ascospores, including several taxa from southeastern Asia, Japan, and Australasia (Gueidan et al. 2014; Gueidan and Lendemer 2015). To accommodate this clade, the genus *Willeya* was resurrected.

Morphologically, *Willeya* is characterized by a crustose, epilithic thallus with a pseudocortex, an association with stichococcoid algae of the green algal genus *Diplosphaera* as photobionts, perithecia containing algal cells in the hymenium (hymenial algae), and muriform ascospores that remain hyaline to pale brownish at maturity. These features distinguish *Willeya* from *Staurothele* s.str., where ascospores become dark brown at maturity, and from *Endocarpon*, which typically exhibits a squamulose or subfruticose thallus (Gueidan et al. 2014; Gueidan and Lendemer 2015).

Currently, 16 species of *Willeya* are recognized worldwide, with a distribution primarily in tropical and subtropical regions, although some species extend into temperate areas. Species of this genus are saxicolous, colonizing a variety of rock substrates, including calcareous rocks, dark-colored aquatic and semi-aquatic rocks, moist vertical riverbanks, and dry siliceous or calcareous surfaces (Goebel 1889; Harada 1992; Gueidan et al. 2014; Aptroot 2016; Orange and Chhetri 2022). Despite this broad distribution, the genus remains poorly known in many regions, and no comprehensive taxonomic revision has been conducted for China. To date, only five species have been reported from China based on scattered records: *W. honghensis* (H. Harada & Li S. Wang) Orange, *W. iwatsukii* (H. Harada) Gueidan, *W. microlepis* (Zahlbr.) Gueidan, *W. pallidipora* (P.M. McCarthy) Gueidan, and *W. tetraspora* Aptroot (Aptroot and Laurens 2003; Harada and Wang 2006; Gueidan et al. 2014; Aptroot 2016; Orange and Chhetri 2022). These records lack detailed morphological documentation and molecular confirmation, leaving the diversity, distribution, and phylogenetic relationships of *Willeya* in China largely unexplored.

In the present study, we aim to fill this gap by conducting a comprehensive taxonomic and phylogenetic assessment of *Willeya* in China. Through morphological examinations, multi-gene (nrITS, nuLSU and mtSSU) phylogenetic analyses and thin-layer chromatography, we report two new species and two newly recorded species from China. Detailed descriptions, illustrations, and comparisons with closely related taxa are provided, along with a worldwide key to all known species of *Willeya*.

## Materials and methods

### Specimen collection and herbarium deposits

Fieldwork was conducted in the Guizhou, Henan, Shaanxi, and Yunnan provinces of China. Fresh specimens of *Willeya* were collected from various rock substrates, air-dried, and subsequently deposited in the Fungarium of the College of Agriculture and Biology, Liaocheng University (**LCUF**) and the Lichen Section of the Botanical Herbarium, Shandong Normal University (**SDNU**). In total, 12 specimens were examined in this study, including newly collected materials and specimens obtained through loans. Voucher data for all specimens used in morphological and molecular analyses are provided in the taxonomy section.

### Morphological and chemical studies

External morphological features were observed using an Olympus SZX16 dissecting microscope (Olympus Corporation, Tokyo, Japan). Anatomical characteristics were examined and photographed with an Olympus BX53 compound microscope equipped with an Olympus DP74 digital camera. Hand-cut sections of perithecia were prepared using a sterile razor blade and mounted in water, 10% potassium hydroxide (K), Lugol’s iodine solution (I), or lactophenol cotton blue (LCB) for observation. Measurements of ascospores, asci, and other anatomical structures were taken from water mounts. Spot test reactions were carried out using K (10% aqueous KOH), C (saturated aqueous sodium hypochlorite) and KC (10% aqueous KOH followed by saturated aqueous sodium hypochlorite). Rock substrate type was assessed using 10% hydrochloric acid (HCl) to test for the presence of carbonates. Secondary metabolites were identified by thin-layer chromatography (TLC) following Orange et al. (2001) and Schumm and Elix (2015).

### DNA extraction, PCR amplification, and sequencing

Genomic DNA was extracted from 3–4 perithecia per specimen using a DNAsecure Plant Kit (Tsingke, Beijing, China), following the manufacturer’s instructions. The final elution volume was 80 μL. Three loci were amplified: the nuclear ribosomal internal transcribed spacer (nrITS) region, including ITS1, ITS2, and the 5.8S rRNA gene; the nuclear large subunit ribosomal RNA gene (nuLSU); and the mitochondrial small subunit ribosomal RNA gene (mtSSU). Primer pairs and thermocycling conditions used for PCR amplification are listed in Table 1. PCR products were purified and sequenced by Tsingke Biotechnology Co., Ltd. (Beijing, China). Newly generated sequences were assembled and edited using Geneious Prime 2025.0.2 (Biomatters Ltd., Auckland, New Zealand; available at https://www.geneious.com), then submitted to GenBank (Table 2).

**Table 1.** Primers and PCR conditions used to amplify nrITS, mtSSU and nuLSU.

**Region**	**Primer pairs**	**References**	**PCR conditions**
ITS	ITS1F/ITS4	Gardes and Bruns (1993) White et al. (1990)	3 min 94 °C, 35 × (30 s 94 °C, 30 s 52 °C, 1 min 30 s 72 °C), 10 min 72 °C.
mtSSU	mrSSU1/ mrSSU3R	Zoller et al. (1999)	5 min 95 °C, 35 × (45 s 95 °C, 1 min 50 °C, 1 min 30 s, 72 °C), 10 min 72 °C.
nuLSU	AL2R/LR6	Vilgalys and Hester (1990)	5 min 94 °C, 35 × (30 s 94 °C, 30 s 56 °C, 1 min 30 s 72 °C), 10 min 72 °C.

**Table 2.** Specimens and sequences for phylogenetic analysis. Newly obtained sequences are in bold; “–” indicates that no sequence was available.

**Taxon**	**Locality**	**Voucher**	**GenBank number (nrITS)**	**GenBank number (nuLSU)**	**GenBank number (mtSSU)**
*Verrucaria viridula*	France	CG 587b (MARSSJ)	KF959786	EF643814	–
*V. weddellii*	France	CG 460 (MARSSJ)	KF959787	EF643812	–
***Willeya bispora***	**China**	**HY251121 (LCUF)**	**PZ344704**	**PZ339023**	–
*W. diffractella*	USA	CG 585 (DUKE)	KF959788	EF643773	–
*W. diffractella*	Canada	Lendemer 28379	KM371614	–	–
*W. diffractella*	USA	Harris 44093	KM371613	–	–
*W. diffractella*	USA	Lendemer 13548	KM371609	–	–
*W. eminens*	Nepal	Orange & Chhetri 18533 (NMW)	OM228811	–	–
*W. fusca*	Vietnam	CG 1912 (BM)	KF959806	KF959823	–
*W. fusca*	Vietnam	CG 1877 (BM)	KF959805	KF959822	–
***W. guizhouensis***	**China**	**20160708 (SDNU)**	**PZ344711**	–	–
***W. guizhouensis***	**China**	**20160718 (SDNU)**	**PZ344712**	–	–
*W. honghensis*	Nepal	Orange & Chhetri 18487 (NM)	OM228783	–	–
*W. irrigata*	Nepal	Orange & Chhetri 18540 (KATH, NMW)	OM228815	OM228815	–
*W. irrigata*	Nepal	Orange & Chhetri 18475 (KATH, NMW)	OM228775	–	–
***W. iwatsukii***	**China**	**HY251117 (LCUF)**	**PZ344714**	–	–
*W. cf. japonica*	Nepal	Orange & Chhetri 18510 (KATH, NMW)	OM228798	OM228798	–
***W. japonica***	**China**	**GZ17070 (LCUF)**	**PZ344713**	**PZ339027**	**PZ339030**
*W. laevigata*	Vietnam	CG 1852 (BM)	KF959807	KF959824	–
*W. nepalensis*	Nepal	Orange & Chhetri 18473 (NMW)	OM228774	–	–
*W. nepalensis*	Nepal	Orange & Chhetri 18472 (NMW)	OM228773	–	–
*W. nepalensis*	Nepal	Orange & Chhetri 18480 (KATH, NMW)	OM228779	OM228779	OM331697
*W. pallidipora*	Vietnam	CG 1927 (BM)	KF959792	KF959813	–
*W. pallidipora*	Vietnam	CG 1926 (BM)	KF959791	KF959812	–
*W. pallidipora*	Vietnam	CG 1908 (BM)	KF959790	KF959811	–
*W. pallidipora*	Vietnam	CG 1941 (BM)	KF959794	KF959820	–
*W. pallidipora*	Vietnam	CG 1948 (BM)	KF959795	KF959821	–
*W. pallidipora*	Vietnam	CG 1865 (BM)	KF959789	KF959819	–
*W. pallidipora*	Nepal	Orange & Chhetri 18478	OM228777	OM228777	–
***W. pallidipora***	**China**	**SQ250758-1 (LCUF)**	**PZ344710**	**PZ339024**	–
*W. protrudens*	Vietnam	CG 1957a (BM)	KF959803	KF959818	–
*W. protrudens*	Vietnam	CG 1945 (BM)	KF959802	KF959817	–
*W. protrudens*	Vietnam	CG 1943 (BM)	KF959801	KF959816	
*W. protrudens*	Vietnam	CG 1940a (BM)	KF959800	KF959815	–
*W. protrudens*	Vietnam	CG 1885 (BM)	KF959798	KF959814	–
***W. protrudens***	**China**	**20160701 (SDNU)**	**PZ344705**	–	–
***W. protrudens***	**China**	**GZ17157 (LCUF)**	**PZ344708**	–	–
***W. protrudens***	**China**	**GZ17169 (LCUF)**	**PZ344709**	–	–
***W. protrudens***	**China**	**SQ250758-3 (LCUF)**	**PZ344706**	**PZ339025**	**PZ339028**
***W. protrudens***	**China**	**SQ250757 (LCUF)**	**PZ344707**	**PZ339026**	**PZ339029**
*Willeya* Species A	Nepal	Orange & Chhetri 18554 (NMW)	OM228823	OM228823	–

### Phylogenetic analyses

Sequences obtained in this study were initially queried against those in GenBank using BLAST (http://www.ncbi.nlm.nih.gov/BLAST/) to confirm their identity and select closely related sequences for phylogenetic reconstruction (Table 2). *Verrucaria weddellii* Servít and *V. viridula* (Schrad.) Ach., both belonging to *Verrucariaceae*, were designated as outgroups. Multiple sequence alignments for each locus were performed using the MAFFT v7.490 plugin implemented in Geneious Prime 2025.0.2 (Katoh and Standley 2013), with settings appropriate for the variability of each locus. We aligned ITS with L-ING-I, nuLSU with G-ING-I, and mtSSU with E-ING-I, keeping all other parameters at default (Shi et al. 2023). The aligned sequences of nrITS, nuLSU and mtSSU were concatenated using the concatenate function in Geneious to produce a three-locus dataset. Phylogenetic relationships were inferred using both maximum likelihood (ML) and Bayesian inference (BI) methods on the CIPRES Scientific Gateway portal (http://www.phylo.org/portal2/; Miller et al. 2010). For the ML analysis, RAxML-HPC2 on ACCESS v.8.2.12 was employed using the locus-specific model partitions, a GTRGAMMA substitution model, and rapid bootstrap analysis with 1,000 replicates (Stamatakis 2014). For the BI analysis, the best substitution models for the three loci were estimated under the Bayesian Information Criterion (BIC) in jModelTest 2.1.10 (Darriba et al. 2012). Based on these estimations, we applied the K80+G model for ITS, K80+I+G for nuLSU, and HKY for mtSSU (Table 3). MrBayes v.3.2.7a was run using two independent runs for 10,000,000 generations with four chains per run and sampling every 1,000^th^ tree (Ronquist et al. 2012). Convergence was assessed using the average standard deviation of split frequencies, a value below 0.01 indicated that the runs had converged. The first 25% of sampled trees were discarded as burn-in. Posterior probabilities (PP) and bootstrap support (BS) values are shown at each node. Nodes with PP ≥ 0.95 and BS ≥ 70% were considered well-supported, whereas unsupported nodes are marked with a hyphen (-). The resulting phylogenetic trees were visualized in FigTree v.1.4.4 and finalized using Adobe Illustrator CC 2019.

**Table 3.** Best partitioning schemes and nucleotide substitution models selected by jModelTest for the concatenated 3-locus dataset.

**Partition**	**Regions**	**Positions**	**Model for Bayes**
Partition 1	nrITS	1–863	K80+G
Partition 2	nuLSU	864–2817	K80+I+G
Partition 3	mtSSU	2818–3547	HKY

## Results and discussion

### Phylogenetic analyses

**Figure 1.**
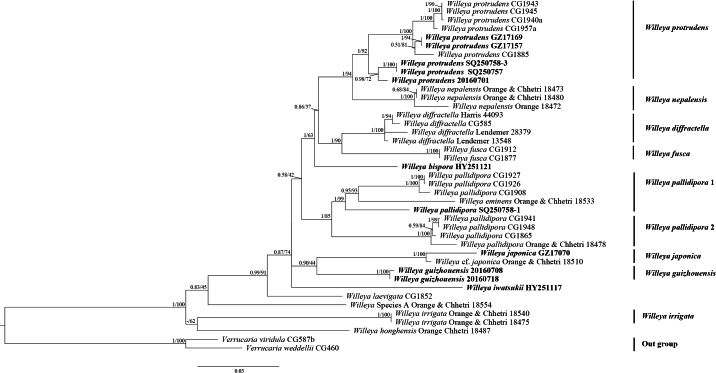
Phylogenetic tree of the genus *Willeya* constructed from ML analyses based on a three-gene dataset (ITS, nuLSU and mtSSU). Support values are on the branches [posterior probability (PP)/bootstrap value (BS)]. The specimens from China are in bold.

We generated 11 new nrITS, 5 new nuLSU, and 3 new mtSSU sequences. Based on these newly obtained sequences together with those retrieved from GenBank, we constructed phylogenetic trees using maximum likelihood (ML) and Bayesian inference (BI) approaches. The ML tree is shown in Fig. 1 and the BI tree yielded a similar topology (Suppl. material 1). The ingroup includes all 11 species of *Willeya* currently available in GenBank (including one unidentified species); we preferentially selected specimens with sequences from multiple loci. Including our newly generated sequences, the ingroup comprises 68 sequences representing 14 species of *Willeya* (Table 2).

In the phylogenetic tree (Fig. 1), all species of *Willeya* formed a well-supported monophyletic clade (BI posterior probability = 1.00, ML bootstrap = 100%). Except for *W. eminens*, which fell within the *W. pallidipora* s. lat. clade, all other 12 *Willeya* species formed distinct lineages.

The two new species, *W. bispora* and *W. guizhouensis*, each formed a distinct, monophyletic clade and were clearly distinguishable from other *Willeya* species based on morphological characters. *W. bispora* differs from all other known *Willeya* species in having two spores per ascus. It formed a clade with *W. diffractella*, *W. fusca*, *W. nepalensis* and *W. protrudens*, receiving strong Bayesian posterior probability (1.00) but moderate to low ML bootstrap support (63%). The two Chinese specimens of *W. guizhouensis* from Guizhou province formed a highly supported clade and were recovered as sister to *W. japonica*, although this relationship received only weak support (PP = 0.90, BS = 44%).

The Chinese specimens of *W. japonica* clustered with the Nepalese specimen in a well-supported clade (PP = 1.00, BS = 100%). *Willeya protrudens* from Guizhou and Shaanxi provinces (China) clustered with the Vietnamese specimens in a well-supported clade (PP = 1.00, BS = 92%) and formed a sister clade to *W. nepalensis*. As in previous studies (Gueidan et al. 2014; Orange and Chhetri 2022), the *W. pallidipora* group was divided into two well-supported and genetically distant subgroups. In our phylogeny, these two subgroups also received high support values (PP = 1.00, BS = 99% and PP = 1.00, BS = 100% respectively). The Chinese specimen from Shaanxi identified as *W. pallidipora* clustered with the lineage referred to as *W. pallidipora* s.lat. by Gueidan et al. (2014), within which the Nepalese specimen of *W. eminens* was nested.

### Taxonomy

#### Willeya bispora

Taxon classification

Fungi

VerrucarialesVerrucariaceae

Xin Yi Wang & X. Zhao sp. nov.

3CDF122A-B1A4-5423-8DB8-AC340718D3C1

Fungal Names: FN 573736

Fig. 2

##### Chinese name.

二孢威氏衣.

**Figure 2.**
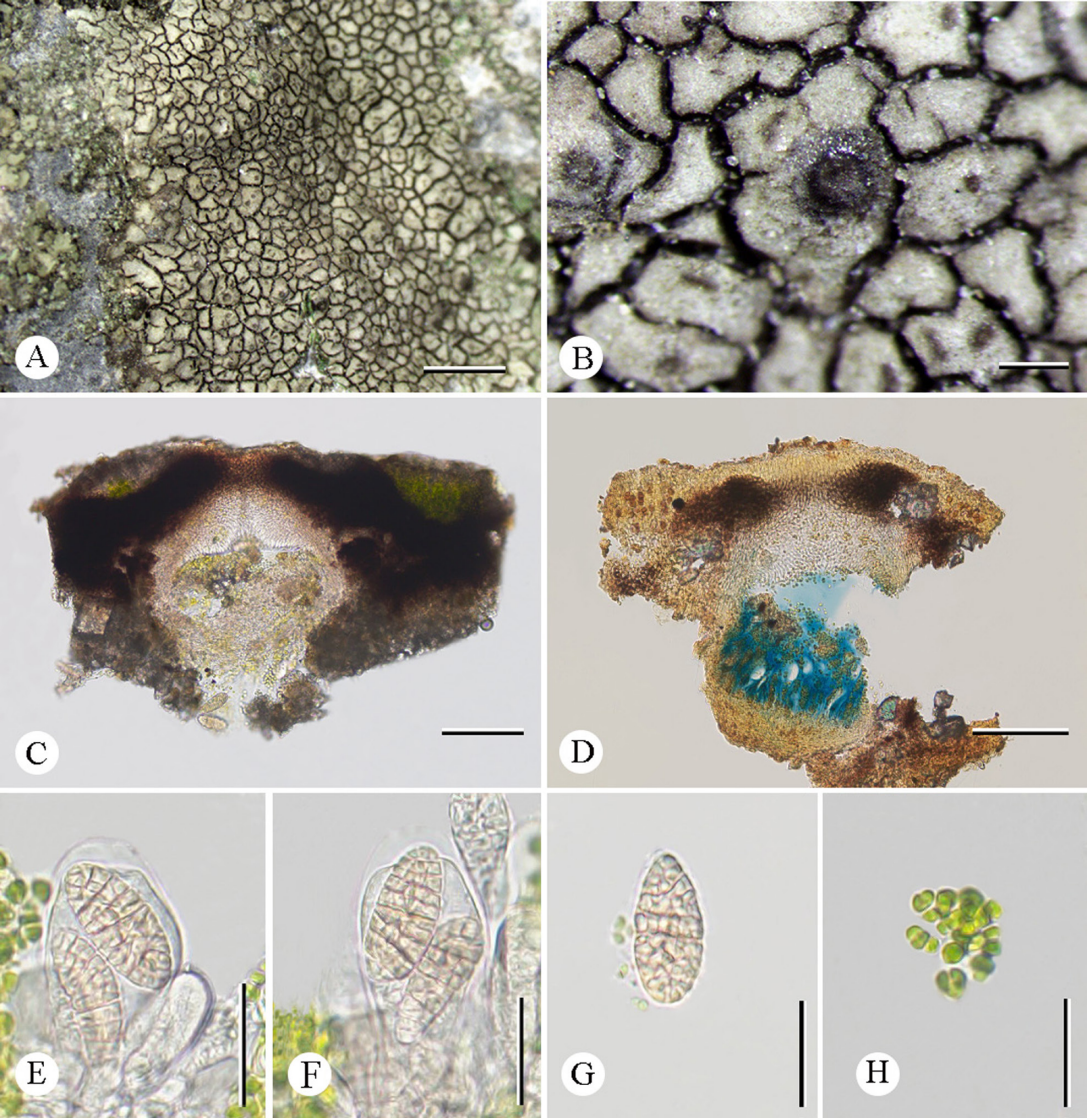
*Willeya bispora* (LCUF HY251121). **A, B**. Thallus with perithecia; **C**. Perithecium section; **D**. Perithecium section in 10% KOH stained with Lugol’s solution; **E, F**. Asci with 2 ascospores; **G**. One ascospore in water. **H**. Algal cells. Scale bars: 1 mm (**A**); 20 µm (**B**); 100 µm (**C**); 50 µm (**D**); 25 µm (**E–H**).

##### Diagnosis.

Distinguished from all other species of the genus by having 2-spored asci (vs. 4–8-spored in other species) and significantly larger muriform ascospores (30.4–41.0 × 13.8–19.9 μm).

##### Type.

China • Henan Province: Xinxiang City, Huixian County, Mount Wanxian, Hanquan, 35°44'40"N, 113°35'09"E, alt. 1080 m, on siliceous rock, 18 July 2025, Wei Li HY251121 (holotype – LCUF).

##### Description.

***Thallus*** crustose, epilithic, continuous, smooth, areolate, pale to olive green, 92.4–98.6 μm thick. Upper cortex 19.2–38.5 μm thick, lower cortex not seen. Medulla poorly defined. The algal layer is 52.2–76.3 µm thick with regularly arranged cells. Black basal layer present. ***Perithecia*** common, scattered over thallus, one perithecium per areole, covered by the thallus, with only the ostiole slightly protruding. ***Ostiole*** brown, 49.9–85.8 μm diam., surrounded by persistent periphyses, 17.9–38.6 μm long. ***Exciple*** brown to colorless. ***Involucrellum*** covering up to half of the apothecium, extending laterally to merge with the black basal layer, 59.3–106.6 μm thick. ***Centrum*** 154–256 μm wide. Hymenial gel I+ red, K/I+ blue. ***Hymenial algal cells*** broadly ellipsoid, 2.5–4.9 × 3.8–5.7 μm, ***Asci*** 2-spored, hyaline, broadly to narrowly ellipsoidal, stipitate, wall thickened above. ***Ascospores*** are muriform, 30.4–41.0 × 13.8–19.9 μm (N = 20). ***Pycnidia*** not seen.

##### Chemistry.

Thallus K–, C–, KC–, UV–. No substances were detected by TLC.

##### Habitat and distribution.

Currently known only from the type locality in Henan Province, China, occurring on siliceous rock in a warm temperate continental monsoon climate.

##### Etymology.

The specific epithet refers to the presence of two spores per ascus.

##### Notes.

This new species was collected from rocks by a spring in Xinxiang City, Henan Province, China, at an altitude of 1080 m. It is primarily distinguished by its 2-spored asci, whereas all other species of *Willeya* have 4–8-spored asci.

In the phylogenetic tree, *W. bispora* forms a sister branch to a clade comprising *W. diffractella*, *W. fusca*, *W. nepalensis* and *W. protrudens*. All four species share green to dark brown thalli, protruding perithecia (either entirely or partly covered by the thallus), and relatively small ascospores. Morphologically, *W. bispora* can be readily separated from these four relatives. *Willeya fusca* has a globose centrum (250–300 µm in diameter), longer hymenial algal cells, 8-spored asci, and smaller ascospores measuring (20–)22–28(–30) × (9–)10–13(–15) µm. *W. diffractella* differs in having perithecia that are immersed with the ostiole either prominent or not, asci with (6–)8 spores, and even smaller ascospores (18–23 × 10–11 µm). *W. protrudens* is characterized by protruding perithecia (entirely or only partly covered by the thallus), 8-spored asci, and smaller ascospores, (20–)22–30(–32) × (9–)10–14(–15) µm. *Willeya nepalensis* bears ascospores of (19.5–)20–21.6–23.5(–25) × (9–)10–10.9–12 µm. Furthermore, *W. bispora* is distinct from *Willeya honghensis* (H. Harada & Li S. Wang) Orange, a species not closely related in our phylogeny. Compared to the latter, *W. bispora* has a thinner thallus (92–99 µm vs. 200–230 µm in *W. honghensis*), a developed involucrellum and broader ascospores (14–20 µm vs. 10–13 µm wide).

#### Willeya guizhouensis

Taxon classification

Fungi

VerrucarialesVerrucariaceae

Xin Yi Wang & X. Zhao sp. nov.

9A2F73DF-39F7-56D9-AB65-0D37B6627DF1

Fungal Names: FN 573737

Fig. 3

##### Chinese name.

贵州威氏衣.

**Figure 3.**
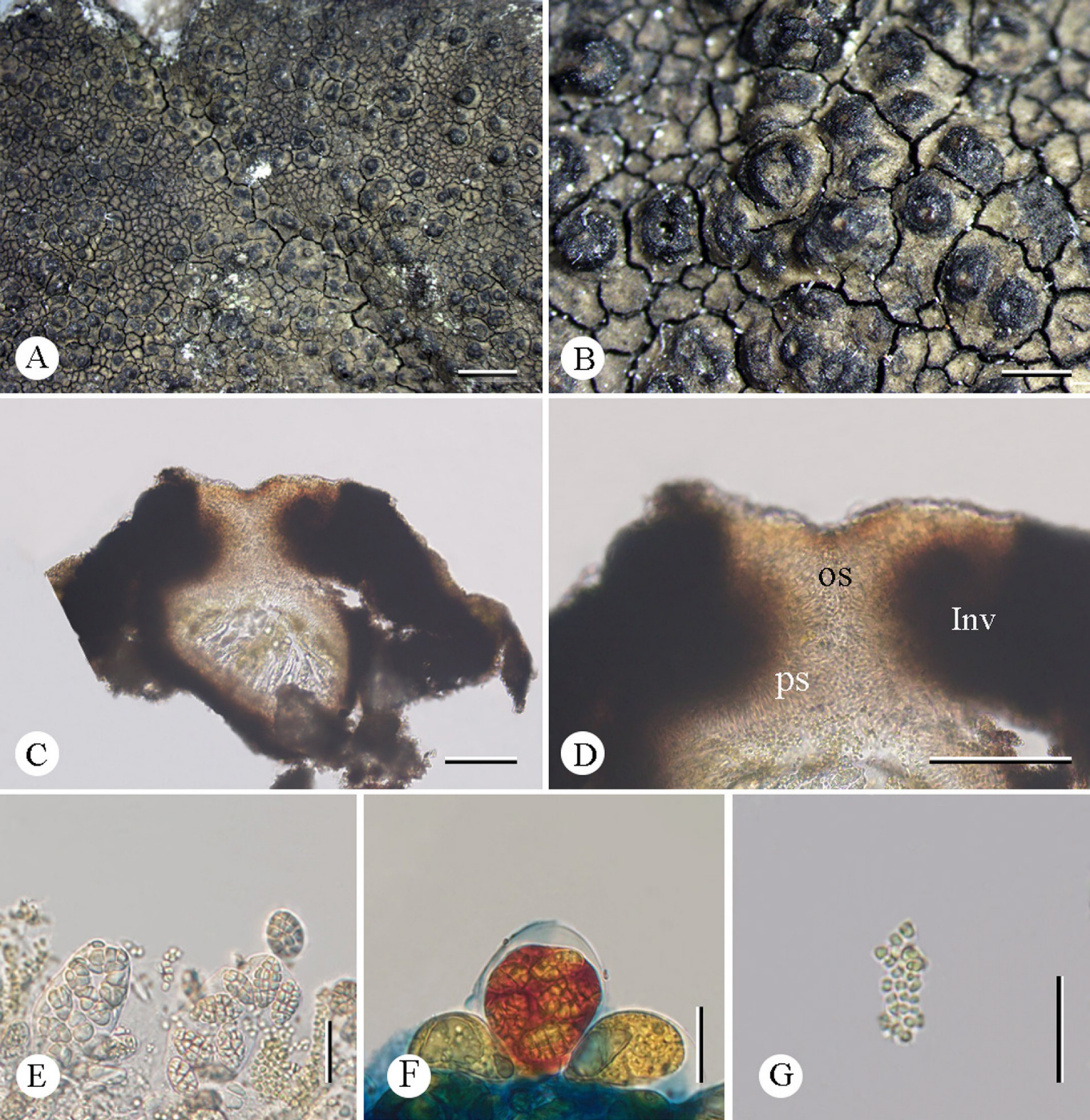
*Willeya guizhouensis* (SDNU 20160718). **A, B**. Thallus with perithecia; **C**. Perithecium section; **D**. Perithecium section showing involucrellum (os: ostiole; inv: involucrellum; ps: periphyses); **E**. Asci, with 8 ascospores; **F**. Asci in 10% KOH stained with Lugol’s solution; **G**. Algal cells. Scale bars: 2 mm (**A**); 50 µm (**B**); 100 µm (**D**); 25 µm (**E, F**); 50 µm (**G**).

##### Diagnosis.

Differing from the most similar species *Willeya diffractella* in possessing 8-spored asci and the presence of black basal layer.

##### Type.

China • Guizhou Province: Jiangkou County, Guanhe Township, Mount Huanggu, 27°34'44.2"N, 108°46'59"E, alt. 900 m, on siliceous rock, 8 April 2016, Wei Cheng Wang & Xiang Xiang Zhao 20160718 (holotype – SDNU).

##### Description.

***Thallus*** crustose, epilithic, greenish gray to medium brown, sparsely dotted with black perithecia, scabrous, matt, rimulose to rimose-areolate, irregular areoles and deeper cracks mostly found around the perithecia, 0.08–0.11 mm thick. Black basal thallus layer present. Algal layer and medulla indistinct. ***Areoles*** 0.2–1.12 mm diam., side of cracks black, black basal layer present. ***Perithecia*** common (1–4 per areole), immersed to half immersed in areoles, forming projections above the thallus level, black perithecial walls exposed, 0.32–1.6 mm wide. ***Ostiole*** visible, 0.04–0.09 mm wide. Periphyses line the inner wall of the ostiole, 47–57.4 μm long. Involucrellum black, spreading laterally and confluent with the excipulum, exciple with dark pigment. ***Centrum*** 268–308 μm wide. Hymenial gel I+ red, K/I+ blue. ***Hymenial algae*** present, globose, 1–4 μm diam. ***Asci*** subglobose to broadly ellipsoid, 8-spored, bitunicate, with the inner wall I+ red, 63.7–84.9 × 30.6–43.3 μm diam. ***Ascospores*** muriform, with 4 transverse septa and 0–3 longitudinal septa, hyaline, I-, 14.8–25.3 × 9.8–13.3 μm (N = 38). ***Pycnidia*** not seen.

##### Chemistry.

Thallus K–, C–, KC–, UV–. No substances were detected by TLC.

##### Habitat and distribution.

On siliceous rock in Guizhou Province, China.

##### Etymology.

The specific epithet guizhouensis refers to the type locality, Guizhou Province.

##### Additional specimens examined.

China • Guizhou Province: Jiangkou County, Guanhe Township, Mount Huanggu, 27°34'44.2"N, 108°46'59"E, alt. 900 m, on siliceous rock, 7 April 2016, Wei Cheng Wang & Xiang Xiang Zhao 20160708 (SDNU).

##### Notes.

Morphologically, this species is characterized by a medium brown to black thallus with a greenish tinge, black perithecia, immersed to half-immersed, forming hemispherical projections, and asci contain eight ascospores. In the phylogenetic tree, *W. guizhouensis* occupies a distinct branch sister to *W. japonica*. The new species can be readily distinguished from its sister species *W. japonica* by the latter’s immersed perithecia (not forming mounds) and thicker thallus (130–290 µm).

Among other morphologically similar species, *W. iwatsukii* differs in having completely immersed perithecia and elongated hymenial algal cells. *W. malayensis* (Zahlbr.) Gueidan is distinguished by elongated hymenial algal cells, the absence of a black basal thallus layer, and narrower ascospores (23–25 × 11–13 µm). *Willeya diffractella* (Nyl.) Müll. Arg. lacks a black basal thallus layer, possesses globose to elongated hymenial algal cells, and has (6–)8-spored asci. *Willeya microlepis* differs in having narrower ascospores (8–10 µm vs. 9.8–13.3 µm), a slightly longer spore length range (18–26 µm vs. 14.8–25.3 µm) and globose hymenial algal cells (2 µm in diameter). Finally, *W. nepalensis* Orange is characterized by a cracked thallus with dark-sided areoles and weakly prominent perithecia (Orange and Chhetri 2022).

#### Willeya japonica

Taxon classification

Fungi

VerrucarialesVerrucariaceae

(B. de Lesd.) Gueidan, Lichenologist 46(4): 529 (2014)

B1CC0095-6F73-5CA6-869C-B26C58470E2B

Fig. 4

##### Basionym.

*Staurothele japonica* B. de Lesd., Bull. Soc. bot. Fr. 68: 494 (1921).

**Figure 4.**
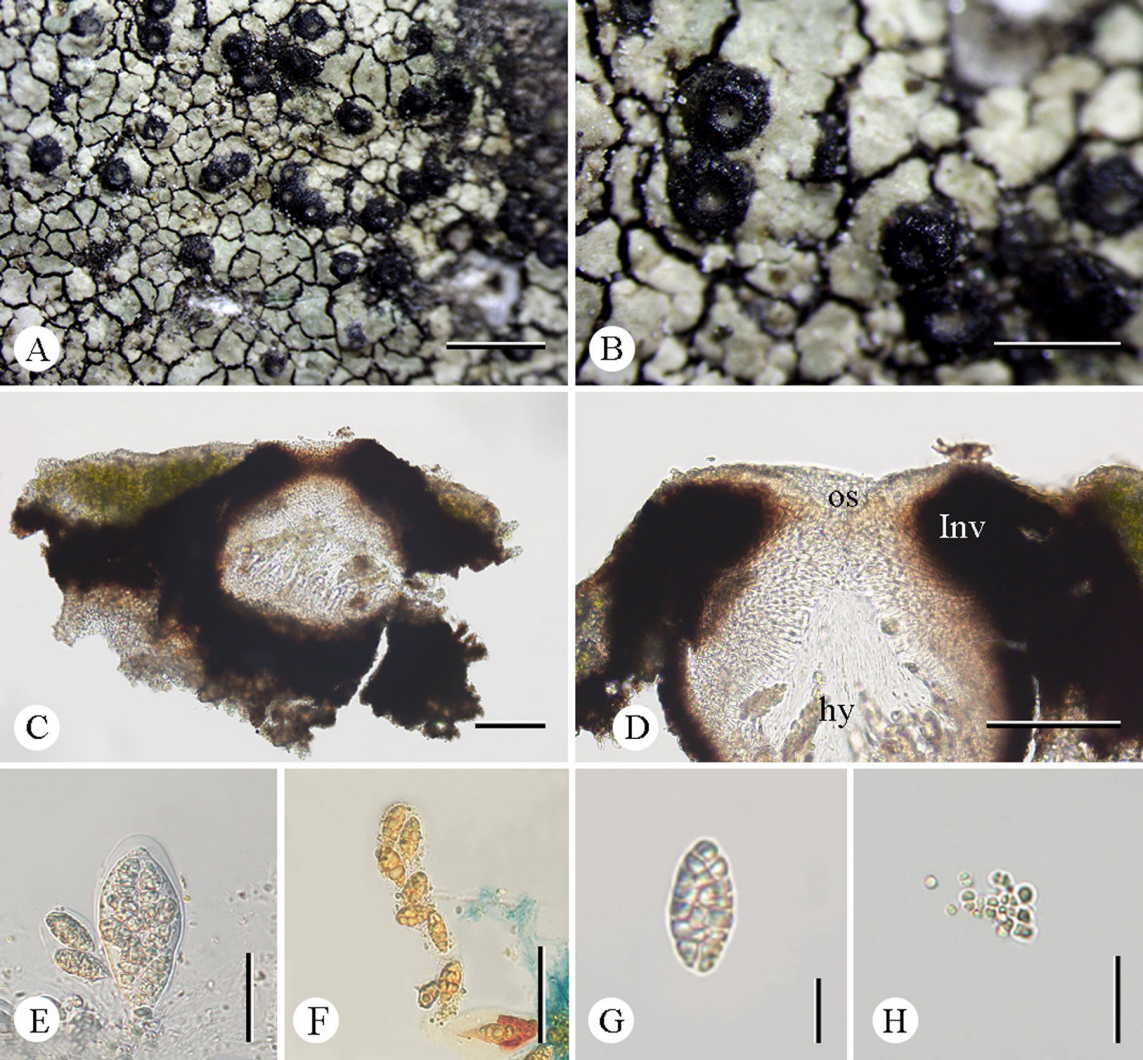
*Willeya japonica* (LCUF GZ17070). **A, B**. Thallus with perithecia; **C**. Vertical section of perithecium; **D**. Perithecium section showing an apical involucrellum. (hy: hymenium; in: involucrellum; os: ostiole); **E**. Asci; **F**. Asci in 10% KOH stained with Lugol’s solution; **G**. One ascospore in water; **H** Hymenial algal cells. Scale bars: 1 mm (**A**); 50 µm (**B**); 100 µm (**C, D**); 50 µm (**E–G**); 25 µm (**H**).

##### Description.

***Thallus*** crustose, epilithic, rimose to areolate, pale to somewhat light brown, 59–87.4 μm thick, with contiguous, varying-sized areoles. Upper cortex paraplectenchymatous, 9–15.8 μm thick. Algal layer uniform, photobiont cells 6.5–12.8 μm diam., regularly arranged, weakly differentiated from the medulla. Medulla inconspicuous. ***Areoles*** 0.2–0.8 mm broad, with fissured sides measuring 0.02–0.04 mm. Black basal layer present. ***Perithecia*** scattered to aggregated, 1–3 per areole, immersed to half immersed, forming projections, not completely covered by the thallus, exposing the ostiole and a blackened platform around it, 244.4–266 μm wide, 303–363.7 μm high. ***Involucrellum*** dark, spreading laterally and extending to the upper half of the perithecium, with cell between the exciple. ***Exciple*** black, 59–93 μm thick. Periphyses present, septate, simple. Paraphyses inconspicuous, hymenium 185.7–220 μm wide, 154.7–255.9 μm long. ***Hymenial algae*** spherical, 2.8–3.6 × 3.9–5 μm diam. ***Asci*** clavate, narrowly elliptical, 84.3–89.5 μm wide, 25.4–27.6 μm high, 8-spored. ***Ascospores*** ellipsoid, muriform, hyaline, 21.9–26.8 × 10.7–12.7 μm (N = 20). ***Pycnidia*** not seen.

##### Chemistry.

Thallus K–, C–, KC–, UV–. No substances were detected by TLC.

##### Habitat and distribution.

On Siliceous rock. Previously known from Japan and Nepal; newly recorded here for China (Guizhou).

##### Specimens examined.

China • Guizhou Province: Xingyi City, Zhengtun Town, Daqing village, Kuzhulin, 25°05'59"N, 105°02'40"E, alt. 1440 m, on siliceous rock, 2 May 2017, Xin Zhao GZ17070 (LCUF).

##### Notes.

The Chinese specimen of *W. japonica* is morphologically highly consistent with the Japanese type (Harada 1992; Gueidan et al. 2014) and the Nepalese specimen (Orange and Chhetri 2022). All share a crustose, areolate thallus, immersed perithecia, 8-spored asci, and muriform ascospores of similar size (Chinese: 21.9–26.8 × 10.7–12.7 μm; Japanese: 21–27 × 8–11 μm; Nepalese: c. 21–24 × 9–14 μm). Minor differences include a thinner thallus in the Chinese material (59–87.4 μm vs. 130–290 μm in Japanese and Nepalese) and slightly broader ascospores. The hymenial algae are spherical in Chinese and Japanese specimens (2–3 μm diam.) but broadly ellipsoid in the Nepalese specimen (2.5–3.3 × 2.5–2.9 μm). An epinecral layer is present in the Japanese type but not observed in Chinese or Nepalese collections. Despite these subtle variations, the overall morphological correspondence is strong. Phylogenetically, the ITS sequences of the Chinese specimens show high similarity (99.46% identity) to the Nepalese specimen, confirming their conspecificity. The Japanese type lacks molecular data, but its morphology aligns well with both Chinese and Nepalese material.

Morphologically, *W. japonica* resembles *W. iwatsukii* and *W. nepalensis*. However, *W. iwatsukii* possesses a rimulose thallus, oblong to bacilliform hymenial algae, and 6–8 spores per ascus. *W. nepalensis* differs in having spherical hymenial algae and relatively shorter and broader ascospores, measuring (19.5–)20–21.6–23.5(–25) × (9–)10–10.9–12 μm.

#### Willeya protrudens

Taxon classification

Fungi

VerrucarialesVerrucariaceae

Gueidan, Lichenologist 46(4): 531 (2014)

8E75E875-EEBB-53AE-8224-C1396C7B664F

Fig. 5

##### Description.

***Prothallus*** conspicuous, grayish green. ***Thallus*** epilithic, continuous, rimose-areolate, grayish-white to light brown. Areoles with 1–3 perithecia, 0.52–1.37 mm diam., surrounded by a fissure 0.01–0.03 mm wide. Cortex 9.7–13 μm thick. The cortex and medulla are not distinctly differentiated. Medulla absent. Dark basal layer present. ***Perithecia*** completely or partially covered by the thallus, forming distinct projections, with an involucrellum that is often only basally covered by the thallus, 256–329 × 470–541 μm. Ostiole apical, conspicuous, surrounded by periphyses. Exciple black, 100–142 μm thick. ***Involucrellum*** is well-developed, merging with the involucrellum downward, laterally spreading. Centrum 250–380 μm wide. Hymenial gel K/I+ blue. ***Hymenial algal cells*** are elongated to cylindrical, septate, 7.3–11.8 × 1.8–2.1 μm in size. ***Asci*** are 8-spored, hyaline, and ellipsoid, wall thickened at the apex, KI+ red, ***Ascospores*** are muriform, colorless, ellipsoid, 21.2–24.2 × 9–12 μm diam (N = 20). ***Pycnidia*** not seen.

**Figure 5.**
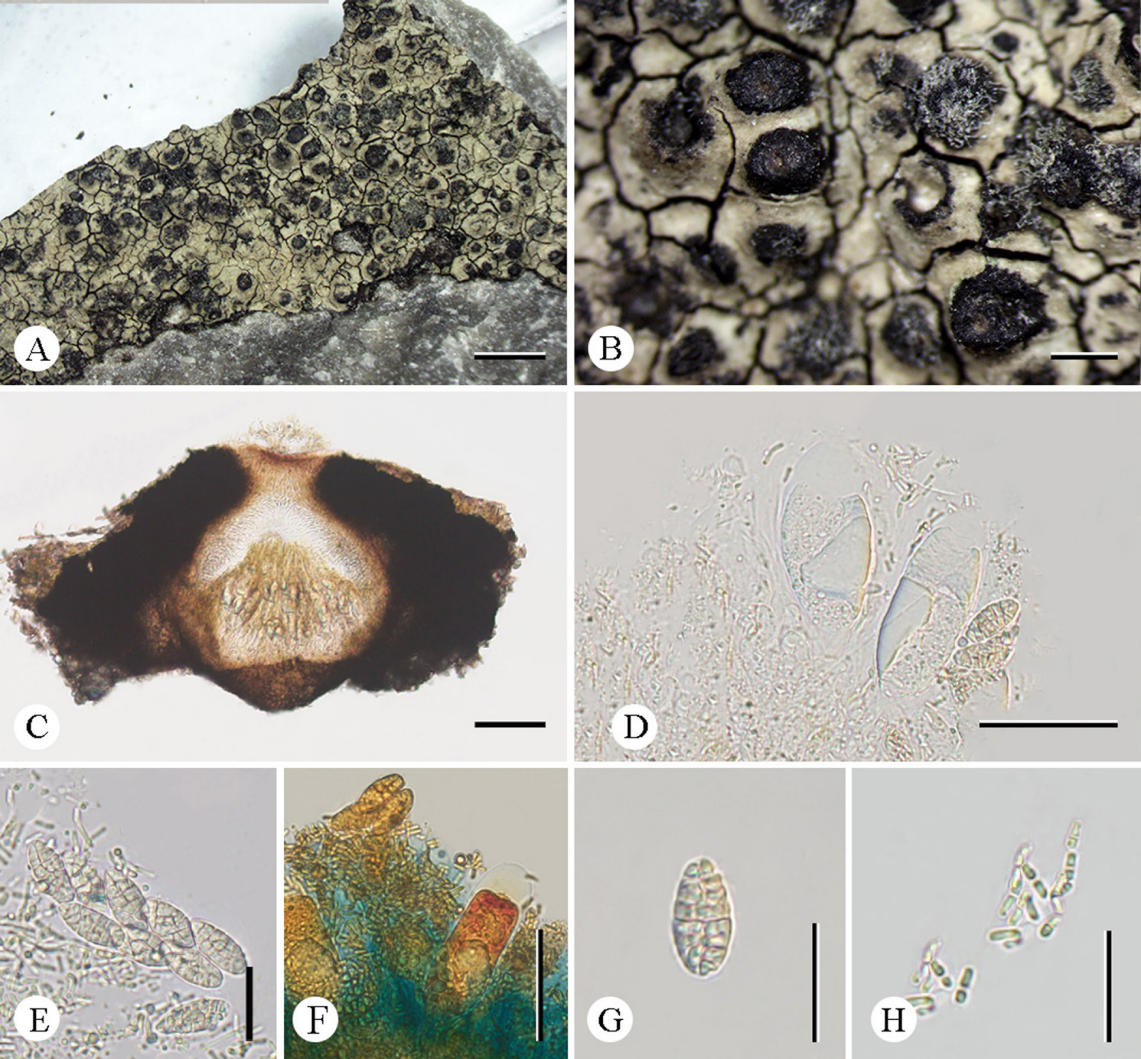
*Willeya protrudens* (SDNU 20160701). **A, B**. Thallus with perithecia; **C**. Perithecium section; **D**. Asci; **E**. Asci with 8 ascospores; **F**. Asci in 10% KOH stained with Lugol’s solution; **G**. One ascospore in water; **H**. Hymenial algal cells. Scale bars: 2 mm (**A**); 0.5 mm (**B**); 100 µm (**C**); 50 µm (**D–F**); 25 µm (**E, G, H**).

##### Chemistry.

Thallus K–, C–, KC–, UV–. No substances were detected by TLC.

##### Habitat and distribution.

On Siliceous rock. Previously known from Vietnam; here newly recorded for China (Guizhou and Shaanxi provinces).

##### Specimens examined.

China • Guizhou Province: Xingyi City, Zhengtun town, Daqing village, 25°05'59"N, 105°02'40"E, alt. 1330 m, on siliceous rock, 2 May 2017, Xin Zhao GZ17157 (LCUF); • Wanfenglin, 24°59'24"N, 104°55'15"E, alt. 1130 m, on siliceous rock, 4 May 2017, Xin Zhao GZ17169 (LCUF); • Shannxi Province: Baoji City, Mount Taibai, Lianhuafeng Waterfall, 34°02'52"N, 107°53'24"E, alt. 1135 m, on siliceous rock, 6 June 2025, Xin Yi Wang SQ250757-1, SQ250757-2, SQ250758-3 (LCUF); • Guizhou Province: Jiangkou County, Guanhe Township, Yuliangxi, alt. 450 m, on rock, Wei Cheng Wang & Xiang Xiang Zhao 20160701 (SDNU).

##### Notes.

*Willeya protrudens* is characterized by a grayish green to olive-brown, rimose to sub-areolate thallus, and perithecia that are entirely or partly covered by the thallus, forming projections above the thallus level. In the multigene phylogenetic tree, all *W. protrudens* specimens form a distinct clade with strong support (PP/BS = 1.00/92%). The type specimen of this species was collected in Vietnam. The Chinese specimens largely match the morphology of the type specimen, except that the latter has a slightly larger centrum (300–400 μm).

###### Species of *Willeya* reported in China

#### Willeya honghensis

Taxon classification

Fungi

VerrucarialesVerrucariaceae

(H. Harada & Li S. Wang) Orange, in Orange & Chhetri, Lichenologist 54(3–4): 166 (2022)

A16856AF-175B-510D-B75B-9D68F8B57AA1

##### Basionym.

*Staurothele honghensis* H. Harada & Li S. Wang, Lichenology 5(1): 15 (2006).

##### Type.

China • Yunnan Province: Hekou County, Manhao town, 13 January 2005, Harada 21262 (CBM-FL-16423–holotype; KUN-L–isotype).

This species was originally described from Yunnan, China (Harada and Wang 2006) and subsequently reported from Nepal (Orange and Chhetri 2022). Although we did not examine specimens of this species directly, its phylogenetic position was inferred from GenBank sequences included in our multigene analysis. Important morphological features of *W. honghensis* include a well-developed, cracked thallus with black-sided areoles, immersed perithecia with only the extreme apex visible, and the occasional presence of pycnidia (Orange and Chhetri 2022). In the protologue, perithecia are depicted as forming rather distinct projecting mounds, though anatomical sections suggest this character is not always well developed (Harada and Wang 2006; Orange and Chhetri 2022). The species is distinct from *W. tetraspora* by its shorter ascospores (Aptroot 2016).

In our phylogenetic tree, *W. honghensis* occupied a basal position within *Willeya*. In the maximum likelihood (ML) analysis, it formed a weakly supported sister relationship with *W. irrigata* (BS = 62%, Fig. 1), whereas in the Bayesian inference (BI) tree (Suppl. material 1), it appeared as a separate lineage, parallel to both the *W. irrigata* clade and the remaining *Willeya* species. This placement is partially consistent with the findings of Orange and Chhetri (2022), who recovered *W. honghensis* as basal to a well-supported clade comprising *Endocarpon* species rather than among other *Willeya* species. The discrepancy between our results and those of Orange and Chhetri (2022) may be due to differences in taxon and gene sampling. Further studies with additional gene regions and broader taxon coverage are needed to resolve the phylogenetic position of *W. honghensis* with confidence.

#### Willeya iwatsukii

Taxon classification

Fungi

VerrucarialesVerrucariaceae

(H. Harada) Gueidan, Lichenologist 46(4): 529 (2014)

F4B11338-D5C0-572C-9F98-F52C81CAD42F

Fig. 6

##### Basionym.

*Staurothele iwatsukii* H. Harada, Nat. Hist. Res. 2(1): 39 (1992).

**Figure 6.**
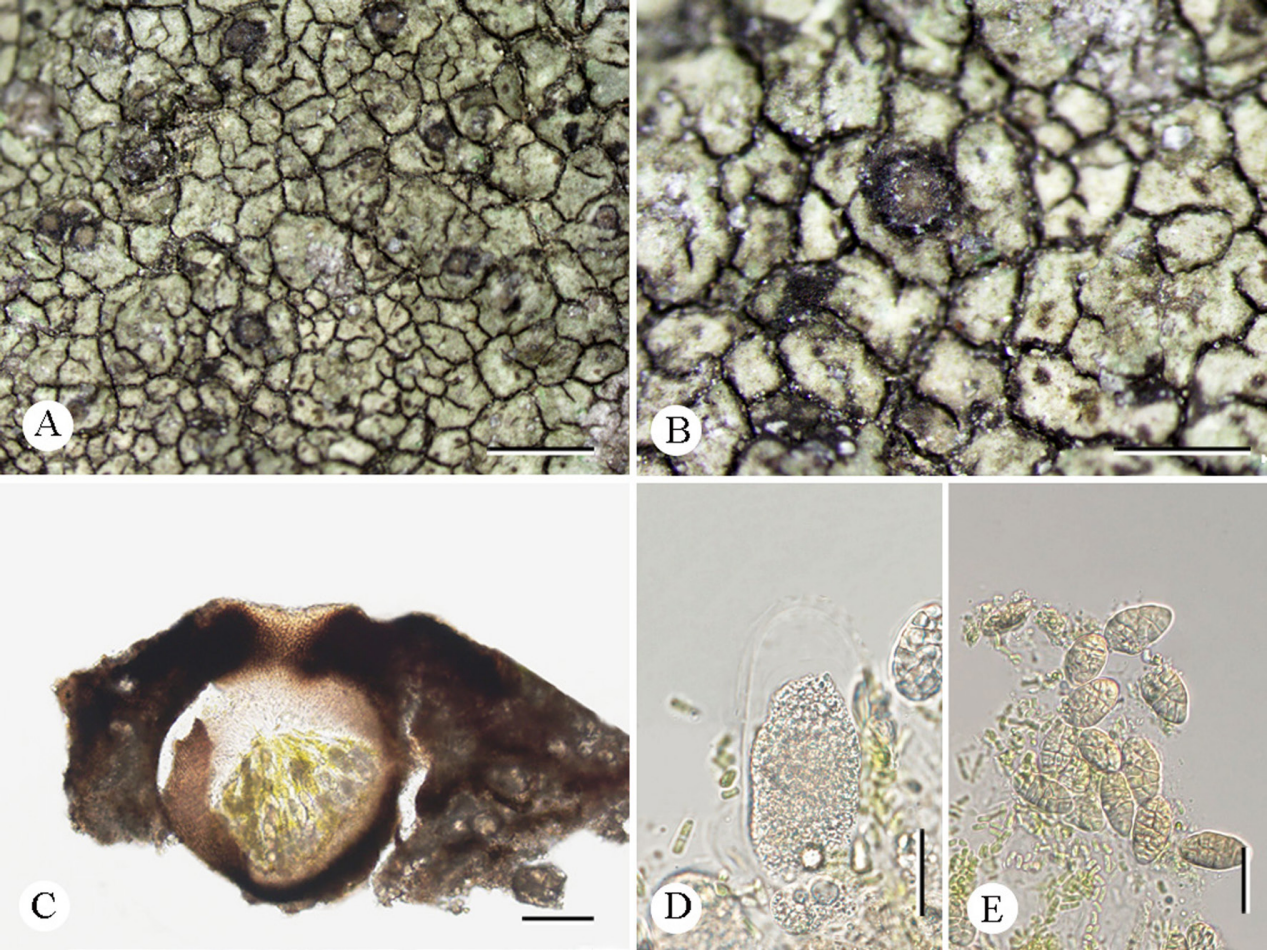
*Willeya iwatsukii* (LCUF HY251117). **A, B**. Thallus with perithecia; **C**. Perithecium section; **D**. Ascus; **E**. Ascospores in water. Scale bars: 1 mm (**A**); 0.5 mm (**B**); 100 µm (**C**); 25 µm (**D**); 25 µm (**E**).

##### Type.

Japan • Shikoku, Kochi-ken, Takaokagun, Yusuhara-cho, josei, alt. 450 m, on rock at the edge of stream, 2 Aug. 1985, H. Harada 3401 (HIRO-holotypus; NMW, CBM-isotypi).

*Willeya iwatsukii* is characterized by a crustose, pale to somewhat brown, rimose to areolate thallus, immersed perithecia (not forming projections), oblong to bacilliform hymenial algae, clavate asci with 6–8 spores, and muriform ascospores. Pycnidia not observed.

Globally, this species was previously known only from Shikoku, Japan (on rocks at the edge of a stream; Harada 1992) and Yunnan Province, China (on rocks beneath trees and on exposed riverside boulders; Harada and Wang 2006). Previous reports noted 8-spored asci and ascospores of 17–24 × 8–12 μm for Chinese material, compared to the 6–8-spored asci and slightly larger spores (20–33 × 8–12 μm) found in Japanese specimens.

Here, we report a new locality from Henan Province, China: Xinxiang City, Huixian County, Mount Wanxian, Hanquan Village (alt. 1080 m), where it was collected on siliceous rock. The newly collected material has 8-spored asci; ascospores are hyaline, muriform, and measure 21.9–31.5 × 10.3–15.0 μm (N = 30; Fig. 6). Chemical spot tests are reported here for the first time: thallus K–, C–, KC–, UV–. No secondary metabolites were detected by TLC.

##### Specimens examined.

China • Henan Province, Xinxiang City, Huixian, Mount Wanxian, Hanquan, 35°44'40"N, 113°35'9"E, alt. 1080 m, on siliceous rock, 17 July 2025, Wei Li HY251117 (LCUF).

#### Willeya microlepis

Taxon classification

Fungi

VerrucarialesVerrucariaceae

(Zahlbr.) Gueidan, Lichenologist 46(4): 530 (2014)

A4BB8DD3-A7EA-580B-A561-FCE8F21ED652

##### Basionym.

*Staurothele microlepis* Zahlbr., in Handel-Mazzetti, Symb. Sinic. 3: 15 (1930).

##### Type.

China • Yunnan Province, Lijiang City, Yongsheng County, alt. 2750 m, 27 June 1914, A. Zahlbruckner 3247 (WU–holotype).

This species was originally described from southwestern China (Zahlbruckner 1930) and to date, is known only from its type locality. No additional collections have been made, and molecular data remain unavailable. *W. microlepis* is morphologically distinct due to its very small areoles (0.2–0.3 mm diam.), minute perithecia (0.2–0.4 mm wide), and ascospores measuring 18–26 × 8–10 µm with a characteristic muriform arrangement (8–10 × 2–4 cells). It resembles *W. japonica* and *W. iwatsukii* in general habit but differs from *W. japonica* in having a thinner thallus (0.2 mm vs. 130–290 µm) and smaller areoles. It differs from *W. iwatsukii* by its rimose-areolate (not rimulose) thallus and globose (not oblong to bacilliform) hymenial algae. The absence of molecular data and recent collections prevents a precise phylogenetic placement; further fieldwork at the type locality is needed to obtain fresh material for molecular analysis and clarify its relationship within *Willeya*.

#### Willeya pallidipora

Taxon classification

Fungi

VerrucarialesVerrucariaceae

(P.M. McCarthy) Gueidan [as ' pallidopora'], Lichenologist 46(4): 530 (2014)

E4E59708-CFED-550F-B4A3-872043B99B75

Fig. 7

##### Basionym.

*Staurothele pallidopora* P. M. McCarthy, Muelleria 8(3): 275 (1995).

**Figure 7.**
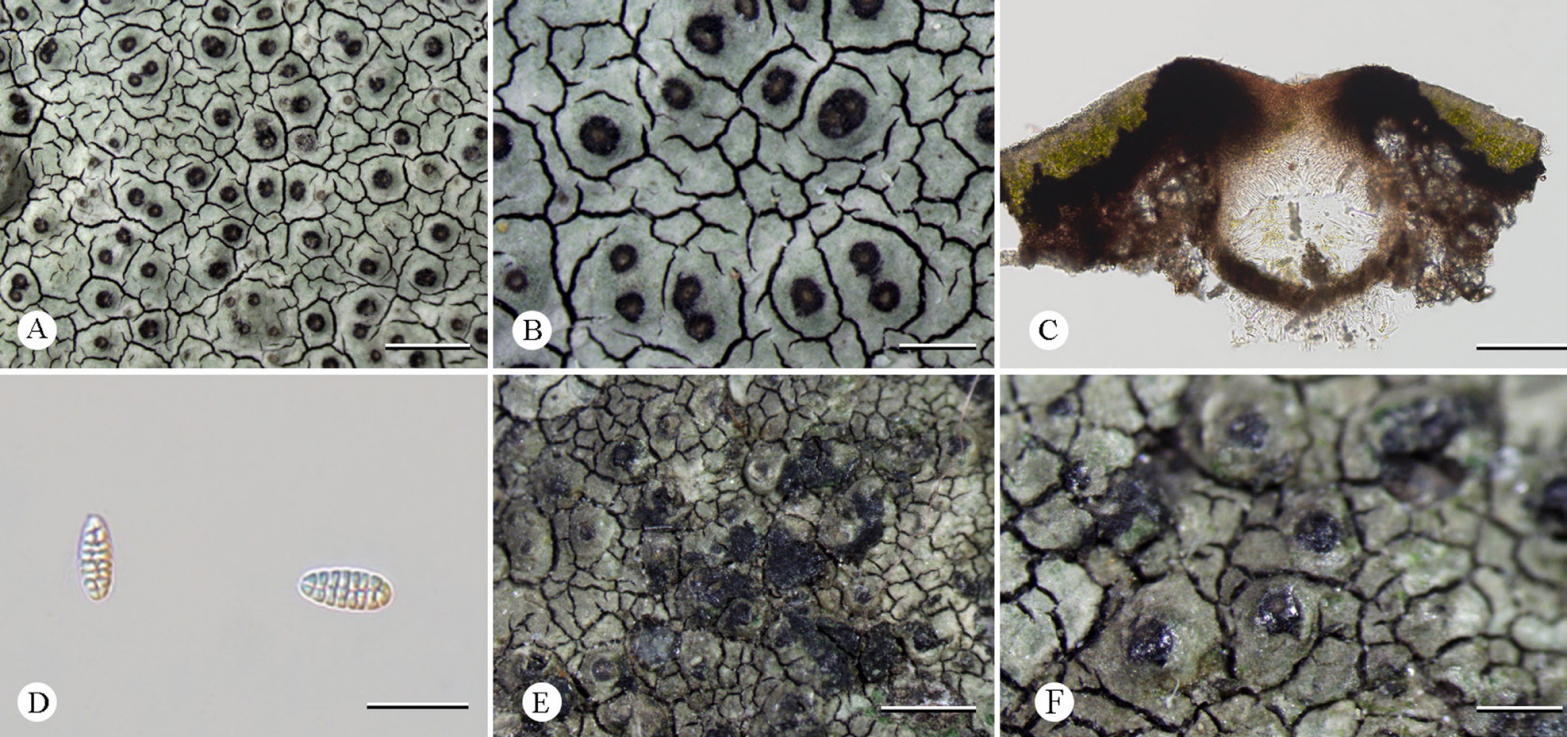
Photographs showing the thallus, perithecium section, and the spores of *W. pallidipora*. **A–D**. YN221805; **E, F**. SQ250758-1. Scale bars: 1 mm (**A, E**); 0.5 mm (**B, F**); 100 µm (**C**); 50 µm (**D**).

##### Type.

Australia • Queensland, Darling Downs district, Bunya Mountains National Park, 50 m below Little Falls, on dry shaded siliceous rocks, 5 September 1993, P. M. McCarthy 768 (MEL–holotype; BRI–isotype).

*Willeya pallidipora* was originally described from Australia (McCarthy 1995) and subsequently reported from Vietnam (Gueidan et al. 2014) and Nepal (Orange and Chhetri 2022). The species exhibits considerable morphological and genetic variation across its range. Gueidan et al. (2014) divided the Vietnamese and Australian specimens into two well-supported, genetically distant subgroups. They noted that morphological and anatomical characters could not clearly distinguish members of the two subgroups, and together with observed morphological variation among the four Australian specimens studied, they concluded that *W. pallidipora* most likely represents a species complex, referred to as *W. pallidipora* s. lat. (Gueidan et al. 2014). Subsequent studies included a Nepalese specimen that clustered with the Vietnamese and Australian specimens, further supporting the complex nature of this taxon (Orange and Chhetri 2022).

In our phylogenetic tree, the Chinese specimen from Shaanxi province clustered within the *W. pallidipora* s. lat. clade (*W. pallidipora* 1, Fig. 1), which also contains the Nepalese specimen of *W. eminens*. This placement suggests that the Chinese specimen may represent a distinct lineage, possibly a cryptic species. However, morphological comparisons with specimens from other regions did not reveal stable diagnostic characters. Specimens from Nepal, Australia, and China share a pale green to dark grey-brown thallus with few cracks, immersed to completely immersed perithecia, and elongate-cylindrical hymenial algae. Ascospore sizes vary among regions: Nepalese specimens 20.5–22.5 × 9.5–11 μm, Australian specimens 18–28 × 9–14 μm, and Chinese specimens 20–24 × 9.7–13 μm (Fig. 7). These overlapping spore dimensions, combined with the lack of clear morphological discontinuities, prevent a reliable separation of the Chinese material from other populations.

Given the high morphological and genetic diversity within the *W. pallidipora* complex and the absence of consistent distinguishing features, we tentatively assign the Chinese specimens to *W. pallidipora* s. lat. Future studies with denser sampling, including additional gene regions and examination of type material across the complex, are needed to resolve the taxonomic status of the Chinese populations.

Chemical spot tests on the thallus yielded negative reactions (K–, C–, KC–, UV–), and no lichen substances were detected by TLC.

##### Specimens examined.

China • Yunnan Province, Mangshi, Mengbanna Xi Exotic Garden, 24°25'44"N, 98°35'19"E, alt. 940 m, on rock, 11 August 2022, Yanfei Zhao YN221805 (LCUF); • Shannxi Province: Baoji City, Mount Taibai, Lianhuafeng Waterfall, 34°02'52"N, 107°53'24"E, alt. 1135 m, on siliceous rock, 6 June 2025, Xin Yi Wang SQ250758-1 (LCUF).

#### Willeya tetraspora

Taxon classification

Fungi

VerrucarialesVerrucariaceae

Aptroot, Herzogia 29(2): 689 (2016)

D4C1E79B-6250-5BB5-9D09-678082229A4B

##### Type.

The Netherlands • Prov. Groningen, Haren, Botanical Garden, Chinese garden, on naturally shaped limestone imported in 1995 from China, 53°11'N, 6°36'E, ca. 10 m alt., 5 May 2016, A. Aptroot 74911 (ABL–holotype).

This species was described from a botanical garden in the Netherlands, where it grows on naturally shaped limestone boulders imported from a karst region in China (Aptroot 2016). Although its natural distribution is presumed to include China, it has not yet been collected here. It represents the first species of *Willeya* reported from Europe and is characterized by 4-spored asci and globose hymenial algae.

Among *Willeya* species with globose hymenial algae, most possess smaller ascospores, with the exception of *W. iwatsukii* (which is typically aquatic and possesses variably globose to elongate hymenial algae). The 4-spored asci distinguish *W. tetraspora* from all other congeners. *Willeya tetraspora* remains morphologically distinct, and its native distribution in China requires future field exploration for confirmation.

### World key to the 18 species of *Willeya*

**Table:** 

1	Ascospores 2 per ascus	***W. bispora***
–	Ascospores 4–8 per ascus	**2**
2	Ascospores 4 per ascus	***W. tetraspora***
–	Ascospores 6–8 per ascus	**3**
3	Mean ascospore length > 29 μm	**4**
–	Mean ascospore length < 29 μm	**5**
4	Ascospores (28.5–)29.5–36(–40) μm long, hymenial algal cells oblong to cylindrical, some at least 3 times as long as wide	***W. irrigata***
–	Ascospores ca. 32–38 μm long, hymenial algal cells broadly ellipsoid	***W. honghensis***
5	Thallus with occasional cracks not delimit areoles, perithecia forming conical-hemispherical projections	***W. eminens***
–	Thallus finely rimose to sub-areolate or areolate, perithecia immersed or not entirely immersed	**6**
6	Thallus continuous, only locally rimose, semi-endolithic centrum 0.4–0.6 mm wide; perithecia completely immersed	***W. laevigata***
–	Thallus finely rimose to sub-areolate or areolate. Centrum 0.2–0.4 mm diam	**7**
7	Hymenial algal cells oblong to cylindrical, some at least 3 times as long as wide	**8**
–	Hymenial algal cells less than 3 times as long as wide	**12**
8	Thallus dark brown to black	***W. fusca***
–	Thallus pale to medium grey, greyish green, yellowish grey, greyish beige, pale brown, or greenish brown	**9**
9	Perithecia projecting above the thallus level, with an involucrellum often only basally covered by the thallus, ascospores (20–)22–30(–32) μm long, pycnidia not seen	***W. protrudens***
–	Perithecia projecting not above the thallus level or not forming mounds	**10**
10	Perithecia almost completely immersed in thallus, not forming mounds or the mounds very low, thallus shabby green or pale grey to pale grey-brown	**11**
–	Perithecia immersed to 1/2 immersed, ascospores 20–23 μm long, pycnidia absent	***W. rimosa***
11	Thallus pale gray to pale or dark gray-brown. Ascospores 18–28 × 9–14 μm	***W. pallidipora***
–	Thallus dull green. Ascospores 19–26 × 11–13 μm	***W. australis***
12	Hymenial algal cells elongated	**13**
–	Hymenial algal cells broadly ellipsoid to globose	**14**
13	Perithecia immersed to slightly raised, forming projections above the thallus level, involucrellum merging with the basal layer of thallus, 5–120 μm thick	***W. iwatsukii***
–	Perithecia immersed to at last not entirely covered by the thallus, involucrellum 200–300 μm thick	***W. malayensis***
14	Thallus dirty brown to dark brown, ascospores 18–26 × 8–10 μm, hymenial algae spherical, ca. 2 μm diam.	***W. microlepis***
–	Thallus pale gray, yellowish brown, greenish gray, or grayish brown, areoles larger or thallus rimose rather than areolate. Ascospores usually wider or longer	**15**
15	Perithecia almost completely immersed, not forming projections, or only the ostiole protruding	**16**
–	Perithecia semi-immersed to prominently projecting, distinctly raised above the thallus surface	**17**
16	Ascospores 21–27 × 8–11 μm, thallus areolate, areoles contiguous to partly dispersed, hymenial algae spherical	***W. japonica***
–	Ascospores (19.5–)20–23.5(–25) × (9–)10–12 μm, thallus rimose, sides of cracks black, with a black basal layer, hymenial algae broadly ellipsoid, 3.7–5.3 × 3.7–4 μm diam.	***W. nepalensis***
17	Hymenial algae spherical, 1–4 μm diam., thallus with a black basal layer, perithecia prominently projecting, 0.32–1.6 mm diam., ascospores 14.8–25.3 × 9.8–13.3 μm	***W. guizhouensis***
–	Hymenial algae ellipsoid to rod-shaped, 3 × 1.5–2.0 μm diam., thallus without a black basal layer, perithecia immersed to semi-immersed, ostiole protruding but perithecial body not distinctly raised, ascospores 15–32 × 9–15 μm	***W. diffractella***

## Supplementary Material

XML Treatment for Willeya bispora

XML Treatment for Willeya guizhouensis

XML Treatment for Willeya japonica

XML Treatment for Willeya protrudens

XML Treatment for Willeya honghensis

XML Treatment for Willeya iwatsukii

XML Treatment for Willeya microlepis

XML Treatment for Willeya pallidipora

XML Treatment for Willeya tetraspora

## Additional information

### Artificial Intelligence (AI) use

The authors accept full responsibility for the content of the manuscript, including the disclosure of any use of AI.

No AI tools were used in the preparation of this manuscript.

### Author ORCIDs

X. Wang https://orcid.org/0009-0004-7658-9138

Y. Wang https://orcid.org/0009-0000-7083-9512

S.D. Leavitt https://orcid.org/0000-0002-5034-9724

W. Zhu https://orcid.org/0009-0002-6972-3947

X. Zhao https://orcid.org/0000-0002-9405-0812

### Data availability

All of the data that support the findings of this study are available in the main text or Supplementary Information.

## Supplementary materials

Supplementary material 1 Phylogenetic tree of the genus *Willeya* Data type pdf Explanation note Phylogenetic tree of the genus *Willeya* constructed from Bayesian analyses based on a three-gene dataset (ITS, mtSSU and RPB2).Posterior probabilities (PP) are shown on the branches. https://binary.pensoft.net/file/1718942This dataset is made available under the Open Database License (http://opendatacommons.org/licenses/odbl/1.0/). The Open Database License (ODbL) is a license agreement intended to allow users to freely share, modify, and use this Dataset while maintaining this same freedom for others, provided that the original source and author(s) are credited. Xinyi Wang, Yuan Wang, Steven D. Leavitt, Wenzhen Zhu, Xin Zhao
