# Supplementary material for: Phylogeny and taxonomy of Willeya (Verrucariales, Verrucariaceae), including two new species and two newly recorded species from China
Source: MycoKeys. 2026 Jul 20;137:21–42. doi: 10.3897/mycokeys.137.197566 (PMC13408571; doi:10.3897/mycokeys.137.197566)
Supplement: Supplementary material 1 — Phylogenetic tree of the genus Willeya [file mycokeys-137-021-s001.pdf]

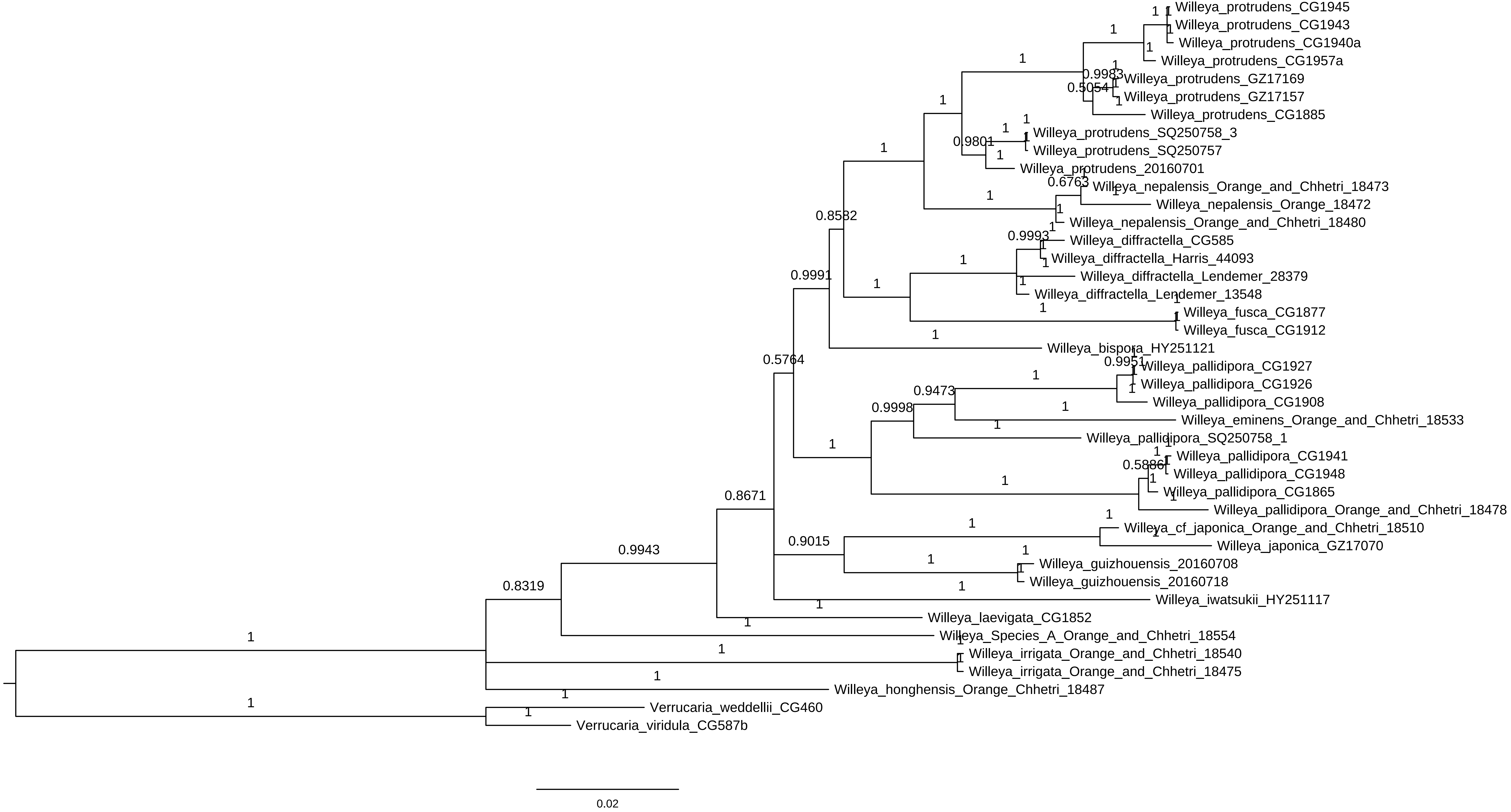

Willeya_protrudens_CG1945
Willeya_protrudens_CG1943
Willeya_protrudens_CG1940a
Willeya_protrudens_CG1957a
Willeya_protrudens_GZ17169
Willeya_protrudens_GZ17157
Willeya_protrudens_CG1885
Willeya_protrudens_SQ250758_3
Willeya_protrudens_SQ250757
Willeya_protrudens_20160701
Willeya_nepalensis_Orange_and_Chhetri_18473
Willeya_nepalensis_Orange_18472
Willeya_nepalensis_Orange_and_Chhetri_18480
Willeya_diffractella_CG585
Willeya_diffractella_Harris_44093
Willeya_diffractella_Lendemer_28379
Willeya_diffractella_Lendemer_13548
Willeya_fusca_CG1877
Willeya_fusca_CG1912
Willeya_bispora_HY251121
Willeya_pallidipora_CG1927
Willeya_pallidipora_CG1926
Willeya_pallidipora_CG1908
Willeya_eminens_Orange_and_Chhetri_18533
Willeya_pallidipora_SQ250758_1
Willeya_pallidipora_CG1941
Willeya_pallidipora_CG1948
Willeya_pallidipora_CG1865
Willeya_pallidipora_Orange_and_Chhetri_18478
Willeya_cf_japonica_Orange_and_Chhetri_18510
Willeya_japonica_GZ17070
Willeya_guizhouensis_20160708
Willeya_guizhouensis_20160718
Willeya_iwatsukii_HY251117
Willeya_laevigata_CG1852
Willeya_Species_A_Orange_and_Chhetri_18554
Willeya_irrigata_Orange_and_Chhetri_18540
Willeya_irrigata_Orange_and_Chhetri_18475
Willeya_honghensis_Orange_Chhetri_18487
Verrucaria_weddellii_CG460
Verrucaria_viridula_CG587b
0.02
